# Analysis of the gene transcription patterns and DNA methylation characteristics of triploid sea cucumbers (*Apostichopus japonicus*)

**DOI:** 10.1038/s41598-021-87278-9

**Published:** 2021-04-07

**Authors:** Lingshu Han, Yi Sun, Yue Cao, Pingping Gao, Zijiao Quan, Yaqing Chang, Jun Ding

**Affiliations:** 1 Key Laboratory of Mariculture and Stock Enhancement in North China’s Sea, Ministry of Agriculture and Rural Affairs, Dalian Ocean University, 52 Heishijiao Rd., Dalian, 116023 Liaoning People’s Republic of China; 2 Ningbo University, Ningbo, 315832 Zhejiang People’s Republic of China

**Keywords:** Biotechnology, Genetics, Molecular biology

## Abstract

Breeding of polyploid aquatic animals is still an important approach and research hotspot for realizing the economic benefits afforded by the improvement of aquatic animal germplasm. To better understand the molecular mechanisms of the growth of triploid sea cucumbers, we performed gene expression and genome-wide comparisons of DNA methylation using the body wall tissue of triploid sea cucumbers using RNA-seq and MethylRAD-seq technologies. We clarified the expression pattern of triploid sea cucumbers and found no dosage effect. DEGs were significantly enriched in the pathways of nucleic acid and protein synthesis, cell growth, cell division, and other pathways. Moreover, we characterized the methylation pattern changes and found 615 differentially methylated genes at CCGG sites and 447 differentially methylated genes at CCWGG sites. Integrative analysis identified 23 genes (such as *Guf1, SGT, Col5a1, HAL, HPS1, *etc.) that exhibited correlations between promoter methylation and expression. Altered DNA methylation and expression of various genes suggested their roles and potential functional interactions in the growth of triploid sea cucumbers. Our data provide new insights into the epigenetic and transcriptomic alterations of the body wall tissue of triploid sea cucumbers and preliminarily elucidate the molecular mechanism of their growth, which is of great significance for the breeding of fine varieties of sea cucumbers.

## Introduction

*Apostichopus japonicus*, which belongs to the *Echinodermata* phylum, is widely distributed in the coastal waters of Russia, Japan, and China^1^. It is an important economic echinoderm worldwide and a pillar industry of the Yellow and Bohai Sea fisheries in China. According to reports, the aquatic production of sea cucumbers in 2017 was 219,907 t, whereas its aquatic production in 2018 was 174,340 t, a decrease of 20.72% compared with the previous year^2^. Due to the disorderly development and blind expansion of the scale of sea cucumber farming, factors such as germplasm degradation, extensive farming, extreme weather, and frequent disease outbreaks have led to miniaturization, low quality (value), and mass death events. This has restricted sea cucumber cultivation and prevented the sustainable development of the aquaculture industry. Germplasm improvement is an important way to enhance the quality of sea cucumbers as well as their growth traits. The practice of breeding has shown that artificial multifold aquatic animals usually have the characteristics of fast growth, large size, and strong stress resistance, which have been widely leveraged in production. Breeding of polyploid aquatic animals remains an important approach and research hotspot for the improvement of aquatic animal germplasm^3^. In terms of echinoderms, Chang Yaqing and others took the lead in the induction of polyploidy in sea cucumbers and achieved success^4^, and then optimized the induction conditions. At present, theoretical research on polyploidy of aquatic animals (especially shellfish, echinoderms, and other invertebrate aquatic animals) is progressing relatively slowly, mostly focusing on the development of induction technology, ploidy detection methods, chromosome karyotype and behavior analysis, and the production, and applications of polyploid animals^5–9^. However, there have been few studies on the genetic characteristics and trait analysis of polyploid aquatic animals at the molecular mechanism level.

DNA methylation is currently the most studied and clearest way of achieving epigenetic modification. The regulation of DNA methylation during gene expression^10–12^ is of great significance to the growth and development of organisms. Hypermethylation of the promoter and coding region can inhibit gene expression, while demethylation of the promoter and coding region can activate gene expression^13–15^. With developments in the field, research on DNA methylation in aquatic animals has become more extensive, but the DNA methylation levels of invertebrate aquatic animals occur at moderate levels. For example, studies have shown that oysters (*Crassostrea Gigas*) have methylated DNA, but the methylation level is lower than that of fish. In oysters, the DNA methylation level of housekeeper genes is higher, and it is speculated that this may be of great significance in inhibiting the transcription of extra promoter regions. DNA methylation plays a regulatory role in oysters, especially in gene families related to stress and environmental response^16^. Osborn et al. believe that epigenetics plays an important role in the regulation of polyploid gene expression^17^, and further evidence has shown that a change in chromosome ploidy is usually accompanied by a change in DNA methylation^18,19^. However, there have been few studies on DNA methylation in triploid aquatic animals. Covelo et al. used a methylation-sensitive amplified polymorphism (MSAP) to evaluate the whole genome methylation changes associated with triploidization in diploid and triploid brown trout (*Salmo trutta*). Statistical analysis of the MSAP data showed that there was no significant difference between diploid and triploid brown trout in the brain, gill, heart, liver, kidney, and muscle samples, which laid a foundation for research on the DNA methylation of triploid aquatic animals^20^. Jiang et al. carried out DNA methylation analysis of a triploid oyster (*C. gigas*) using F-MSAP technology, and showed that some methylation sites of diploid and triploid oysters were ploidy specific, and the mutation rates of methylated sites and unmethylated sites in triploid oysters were higher than those in diploid oysters^21^. They also found that the *CGer* gene was highly expressed in triploid oysters, and its methylation rate was also high, which suggested that *CGer* might play a role in the sterility of triploid oysters^22^.

Transcriptome analysis is widely used in researching biological growth, disease mechanisms, and molecular breeding. With the rapid development of RNA-seq technology, it has been widely used to study gene expression regulation and gene screening of echinoderms, especially the sea cucumbers. In 2012, Du et al.^23^ constructed a cDNA library from different developmental stages and adult tissues of sea cucumbers (*A. japonicus*)*.* Through sequencing and GO and KEGG enrichment analysis, candidate genes involved in metabolism, detoxification, tissue protection, and other pathways and those related to dormancy were selected. In 2014, Zhao et al.^24^ constructed the intestinal cDNA library of sea cucumbers in the normal state, deep dormancy state, and dormancy recovery state, and found that most of the differentially expressed genes (DEGs) in the deep dormancy state were enriched in the metabolism and signal transduction pathways. Chatchaiphan et al. studied the liver transcriptomes of diploid and triploid catfish (*Clarias macrocephalus Günther*) and found that the sequences of diploid and triploid catfish were highly similar, and most gene expression levels were the same^25^. Zeng et al. sequenced the DNA from muscle tissues of diploid and triploid oysters, and found that there were 2045 differential genes, and 28 differential genes expressed only in triploid oysters^26^.

To date, there have been no reports on the DNA methylation pattern and gene expression pattern of the triploid sea cucumbers. Researchers usually thought the growth advantage of triploid aquatic organisms is that the energy of growth is used by reproduction or speculated that the reason for the larger individual of polyploid animals is the doubling of chromosomal material and the increase of cell volume, resulting in larger individuals^27,28^. However, there must be a genetic basis for the differences in traits, and these explanations do not give a definitive answer that relates to the underlying molecular mechanism (gene expression and regulation). Hence, this study aimed to investigate the molecular mechanism of the growth of the triploid sea cucumbers, with the triploid sea cucumbers as the research object and its sibling diploid sea cucumbers as the control. RNA-seq and MethylRAD-seq techniques were used to obtain the expression profiles of body wall tissue DNA and genomic DNA methylation profiles of triploid sea cucumbers to identify the “dosage effect” of gene expression, screen DEGs and differentially methylated genes, and analyze the correlation between gene expression and apparent regulation of triploid sea cucumbers, and finally analyze the molecular mechanism of the growth of triploid sea cucumbers. This study lays a theoretical foundation for the analysis of polyploid echinoderm traits, gene expression, and regulation of the echinoderm, enriches the content of echinoderm research, and provides basic data for the epigenetic study of sea cucumbers.

## Results

### Ploidy detection of triploid sea cucumbers

**Figure 1:**
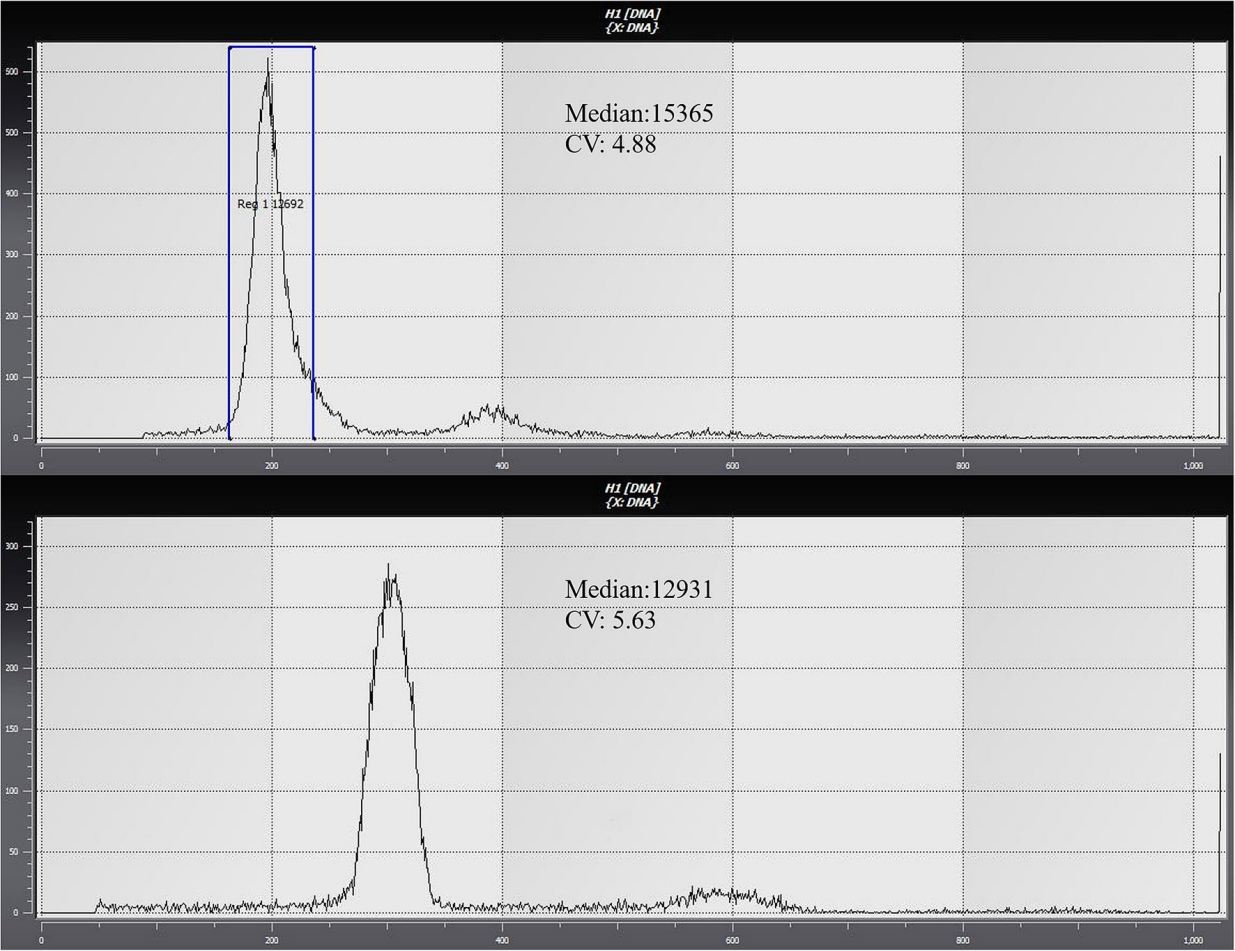
Ploidy test results of diploid sea cucumbers (**A**) and triploid sea cucumbers (**B**).

With diploid sea cucumbers as the control, samples were tested for ploidy via flow cytometry (Sysmex, Japan), the peak value is around 200 for the control diploid sea cucumbers. Compared with the diploid, the triploid sea cucumbers peak should be around 300, as shown in Fig. 1. Through testing, it was found that the triploid inducing rate of sea cucumbers reached 60–80%. Therefore, triploid sea cucumbers were selected and raised.

### Transcriptomic analysis of the body wall tissues of triploid sea cucumbers

**Figure 2:**
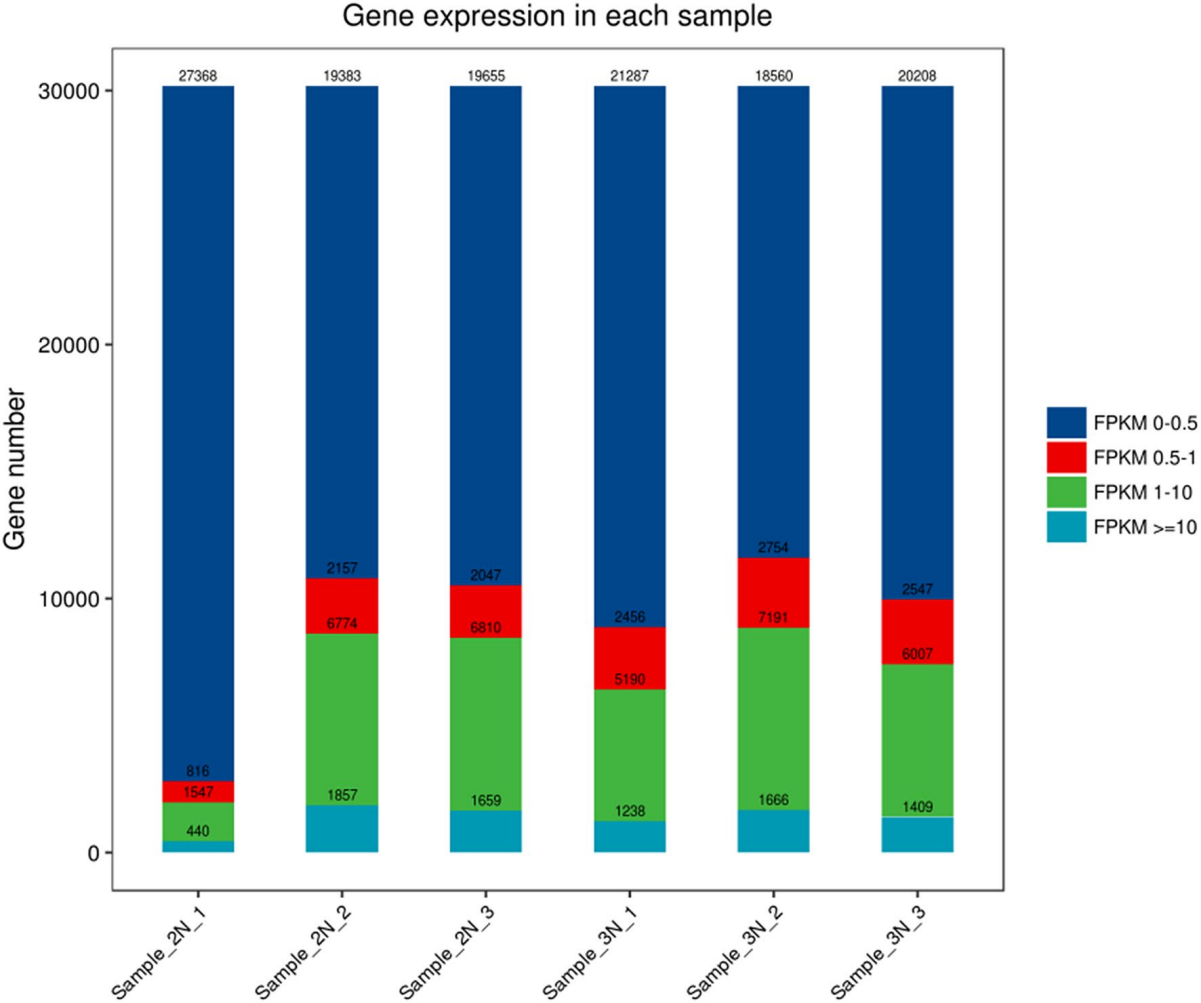
Distribution of FPKM expression. The horizontal axis represents the sample name, the vertical axis represents the number of protein-coding genes, and different colors represent different ranges of FPKM.

After the transcriptome data were extracted, raw reads of normal and triploid sea cucumbers were obtained, which totaled 159.27 M and 169.02 M, respectively. After quality control, clean reads were obtained, which were 21.98G and 23.72G. The base quality value reflects the probability of base sequencing errors. In this study, normal sea cucumber transcriptome had a value of Q30 ≥ 91.94% and triploid sea cucumber transcriptome had a value of Q30 ≥ 93.37%. The specific sequencing data are shown in Table S1, it was found that except for the 2 N-1 sample with a lower comparison rate, the comparison rates of the other 5 samples were all between 64.21 and 76.27%, and the ratios of reads to the unique position of the genome were all more than 58.22% (Table S2). The amount of gene expression is measured by the FPKM value. Data analysis showed that there were no significant differences among the numbers of genes expressed in the six samples; that is, the participation of triploid sea cucumbers and diploid sea cucumbers exhibited no significant “dosage effect” (Fig. 2).

The body wall tissue of diploid sea cucumbers was used as the control group, and the body wall tissue of triploid sea cucumbers was used as the experimental group. The default conditions for screening differences were *p*-value < 0.05 and Fold Change > 2. Transcriptome analysis showed that a total of 640 DEGs were screened, including 337 up-regulated genes and 303 down-regulated genes.

**Figure 3:**
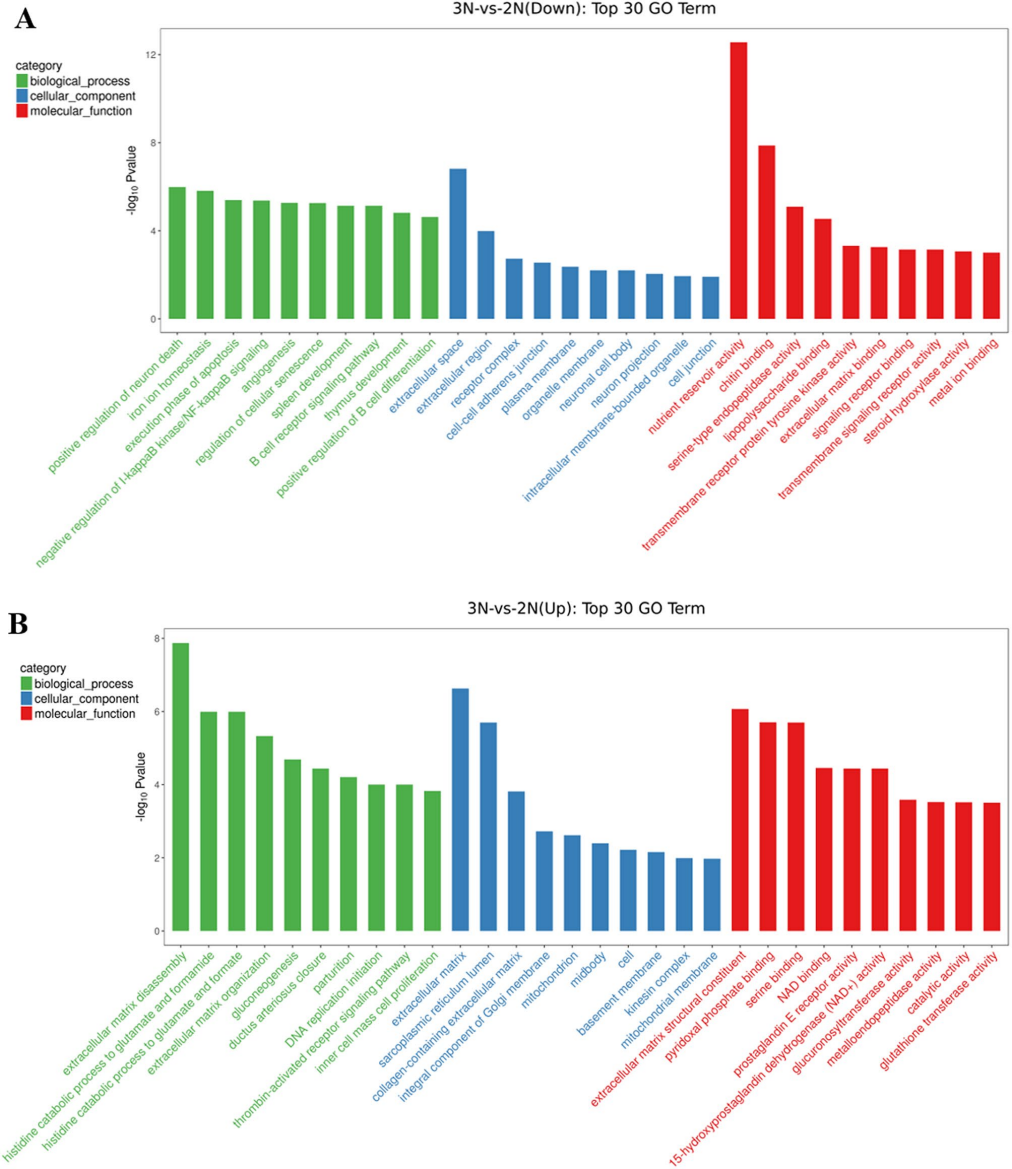
GO enrichment analysis of the top 30 differentially expressed genes. The X-axis is the GO entry name and the Y-axis is -log10 *p*-value (**A**: down-regulated DEGs; **B**: up-regulated DEGs).

The results of GO enrichment analysis showed that 392 DEGs were enriched in 1894 GO entries, of which 1158 were significantly enriched (*p*-value < 0.05). These included 761 GO entries of biological processes, 108 GO entries of cell composition, and 289 GO entries of molecular function. The up-regulated genes were significantly enriched in 774 GO entries (*p*-value < 0.05), including 451 biological processes, 107 cell composition, and 216 molecular functions GO entries; the down-regulated genes were significantly enriched in 768 GO entries (*p*-value < 0.05), including 544 biological processes, 75 cell composition, and 149 molecular functions GO entries. The GO entries with more than 2 DEGs were screened from the three categories (biological process, cell composition, and molecular function), and the corresponding-log10 *p*-value of each entry was sorted from large to small, and 10 entries were selected for mapping (shown in Fig. 3). The results showed that the cell differentiation process had the largest number of DEGs among the biological processes with 18 DEGs. The most significant were the extracellular matrix decomposition (ECM) process, with six DEGs, followed by the regulation of the extracellular matrix organization process, with 3 DEGs. In the cell composition category, the most significant number of DEGs was in the cytoplasmic matrix, with 16 DEGs; the second was collagen type V trimer, with 2 DEGs. Among the molecular functions, chitin binding and transmembrane signaling receptor activity were the most common, with 9 DEGs; the most significant was nutrition reservoir activity, with 5 DEGs, followed by serine binding, with 4 DEGs.

**Figure 4:**
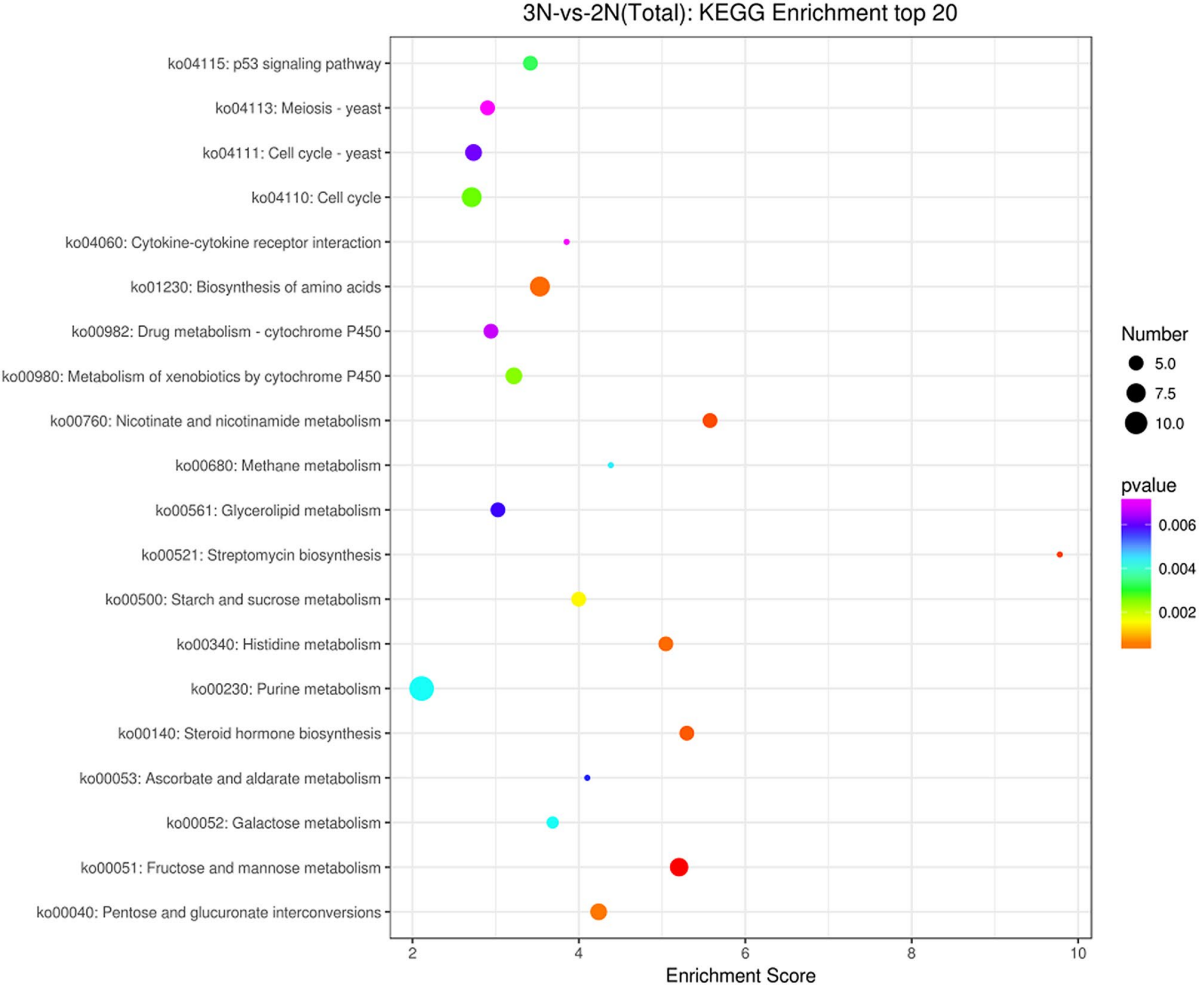
Top 20 differentially expressed genes in the KEGG enrichment analysis. The X-axis is the enrichment score. The larger the bubble, the more differential protein-encoding genes contained in the item. The color of bubbles changes from purple to blue to green to red, with the smaller *p*-value corresponding to greater significance.

The results of KEGG enrichment analysis showed that 418 DEGs were enriched in 170 KEGG signaling pathways, among which 54 KEGG signaling pathways were more significant (*p*-value < 0.05), and the purine metabolism pathway was the most abundant with 12 genes. The up-regulated genes were significantly enriched in 59 KEGG signaling pathways including amino acid synthesis, fructose metabolism, and nicotinamide metabolism (*p*-value < 0.05), such as *Amy* (amylase), *HK* (hexokinase), *UGT* (glucuronosyltransferase), *PGM* (phosphoglucomutase), *GMPP* (guanyltransferase mannose phosphate), *NNT* (nicotinamide nucleus), and other genes. The down-regulated genes were significantly enriched in 37 KEGG signaling pathways (*p*-value < 0.05), including cytokine receptor interaction, the nod receptor signaling pathway, and apoptosis (*p*-value < 0.05), such as *sFLT1* (FMS-like tyrosine kinase 1), *KDR* (kinase insert domain protein receptor), and other genes. The results are shown in Fig. 4.

### DNA methylation analysis of body wall tissues of triploid sea cucumbers

The DNA library was constructed using Methyl RAD technology and sequenced on the Illumina platform. The enzyme reads obtained through quality control were aligned to the constructed CCGG/CCWGG site reference sequence. The comparison rate of enzyme reads to the unique position of the reference sequence was 45.13–46.75%, as shown in Table S3. The sequencing depth of methylation sites (CCGG sites and CCWGG sites) in each sample was counted (Table 1). The average number of CCGG methylation sites was 77,027 for diploid and 76,873 for triploid sea cucumber tissues. The average number of CCWGG methylation sites was 12,448 for diploid and 12,697 for triploid tissues.

**Table 1:** Coverage depth of methylation sites.

Sample name	CCGG		CCWGG	
	Number of sites	Depth	Number of sites	Depth
2 N-1-1	78,479	79.87	12,993	54.02
2 N-2-1	74,606	95.58	10,687	71.44
2 N-3-1	77,997	78.53	13,664	47.10
3 N-1-1	77,076	78.70	13,587	47.24
3 N-2-1	76,711	81.79	13,092	50.66
3 N-3-1	76,832	88.68	11,412	57.91

**Figure 5:**
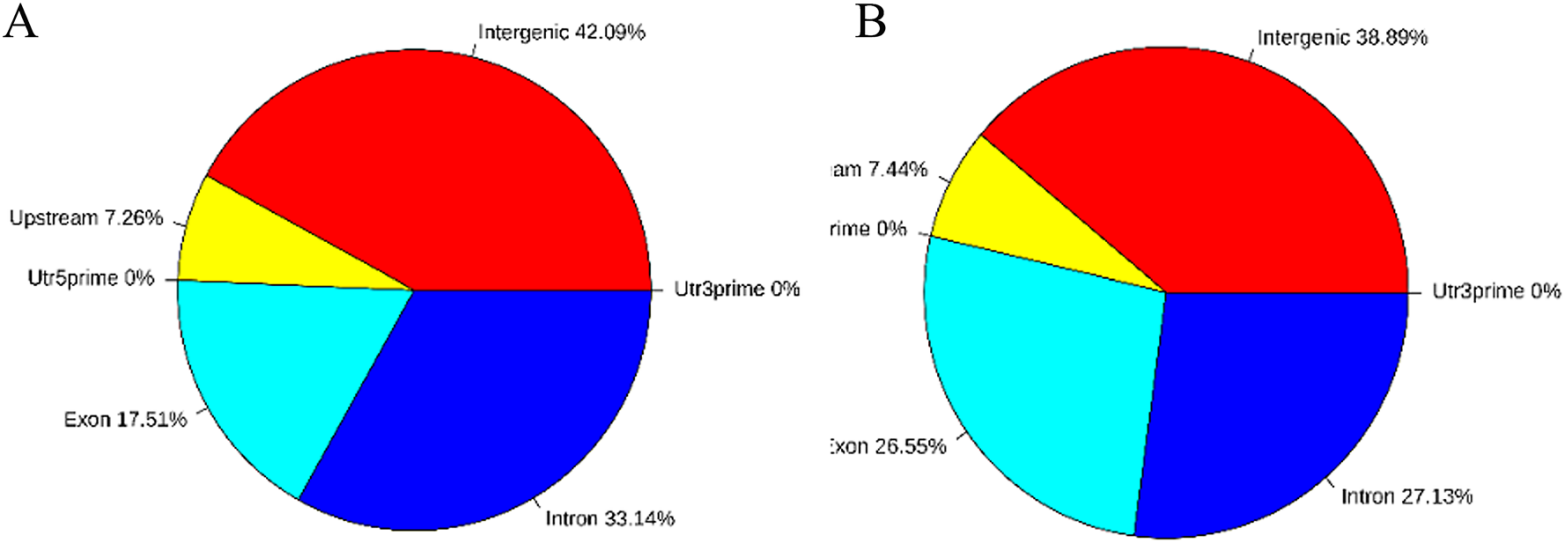
Pie charts of the distribution of the differentially methylated sites on different functional components (**A**: CCGG sites; **B**: CCWGG sites).

Using BEDTools software (v2.25.0), we found that the CCGG methylation sites in diploid and triploid tissues were concentrated in the gene region, followed by the introns, and there was no significant difference between diploid and triploid samples. The CCWGG methylation sites in diploid and triploid tissues were mainly found in the gene region and exon region. CCGG and CCWGG sites were not distributed in untranslated regions (Fig. 5).

**Figure 6:**
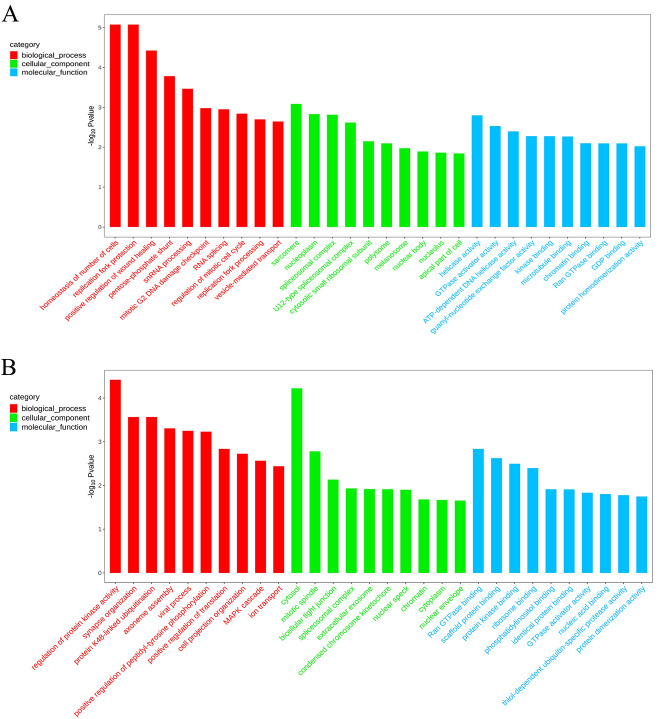
GO functional classification histograms of differentially methylated genes, the X-axis is the GO entry name and the Y-axis is -log10 *p*-value. (**A**: CCGG sites; **B**: CCWGG sites).

The genes with statistically different *p*-value ≤ 0.05 and |log_2_FC|> 1 were screened. A total of 615 differentially methylated genes were screened at CCGG sites, including 325 up-regulated genes and 190 down-regulated genes; 447 differentially methylated genes were screened at CCWGG sites, including 292 up-regulated genes and 155 down-regulated genes. GO enrichment analysis of the differentially methylated genes showed that the most significant enrichments in biological processes were in homeostasis of cell number, replication fork protection mechanism, and positive regulation of wound healing. Among them, the RNA splicing process had the most common differentially methylated genes, and there were 10 differentially methylated genes. In the process of cell composition, sarcomere, nucleoplasmic structure, and spliceosome were significantly enriched. Among them, the term with the most differentially methylated genes was the nucleocytoplasmic structure with 48 differentially methylated genes. Among the molecular functions, the most significant enrichment was in enzyme activity. Among them, the most differentially methylated genes were involved in protein homodimerization activity, and there were 18 differentially methylated genes (Fig. 6).

**Figure 7:**
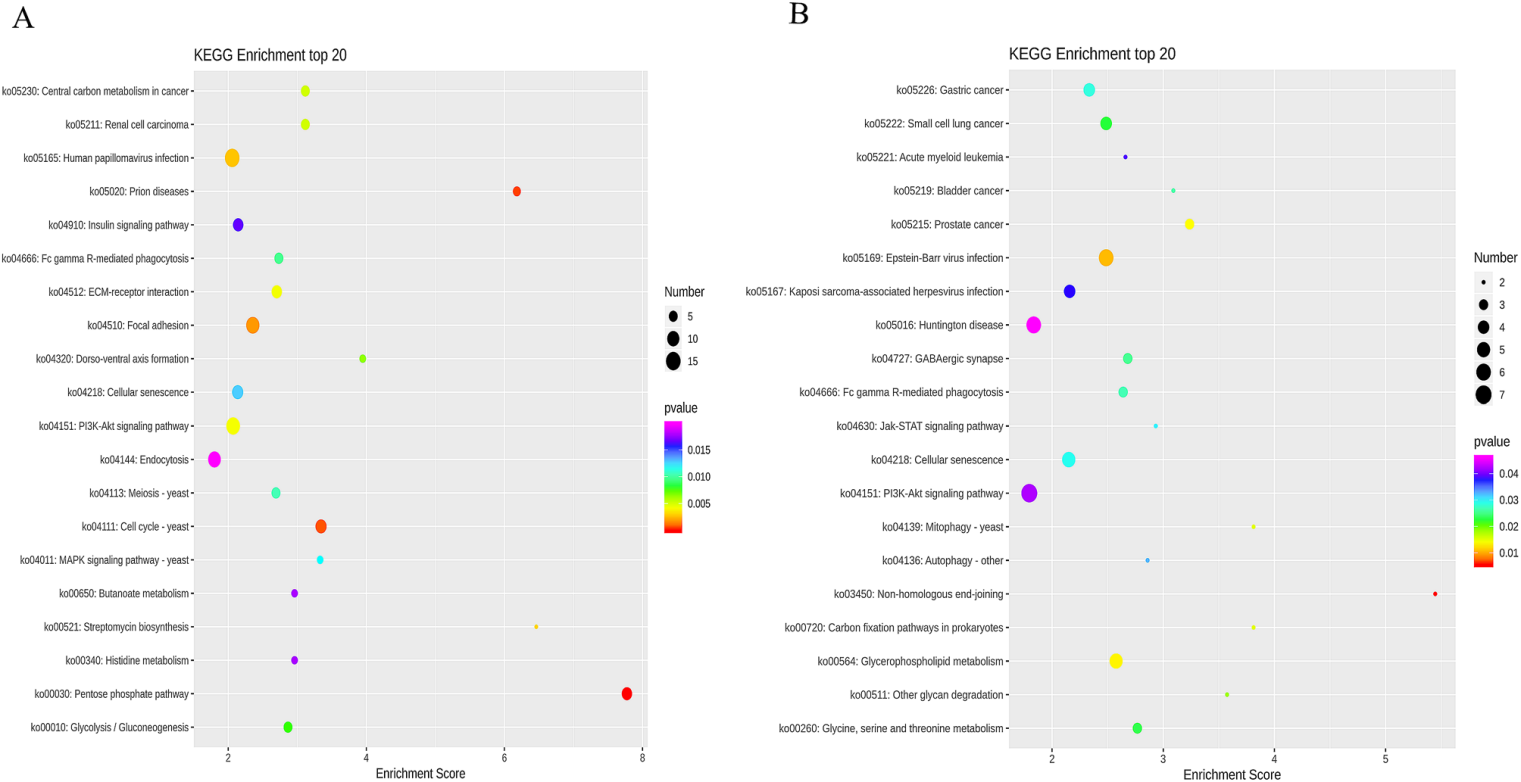
Bubble map of KEGG top 20 differentially methylated genes in CCGG sites (**A**) and CCWGG sites (**B**).

The results of KEGG enrichment analysis showed that differentially methylated genes at CCGG sites were significantly enriched in pathways such as the pentose phosphate pathway, cell cycle, and disease. The most enriched pathway was the human papillomavirus infection pathway with 15 differentially methylated genes. Up-regulated differentially methylated genes were significantly enriched in pathways such as disease, dorsoventral axis formation, and cell aging, and down-regulated differentially methylated genes were significantly enriched in pathways such as the pentose phosphate pathway and the central carbon cycle. Differentially methylated genes at CCWGG sites were significantly enriched in pathways such as heterologous end joining, virus infection, and glycerophosphate metabolism. The most enriched pathway was the PI3K-Akt signaling pathway, with 7 differentially methylated genes. Up-regulated differentially methylated genes were significantly enriched in amino acid metabolism and autophagy, and down-regulated differentially methylated genes were significantly enriched in phagocytosis, sugar synthesis, and lipid metabolism pathways (Fig. 7).

### GO, KEGG annotation and key genes identitication

**Figure 8:**
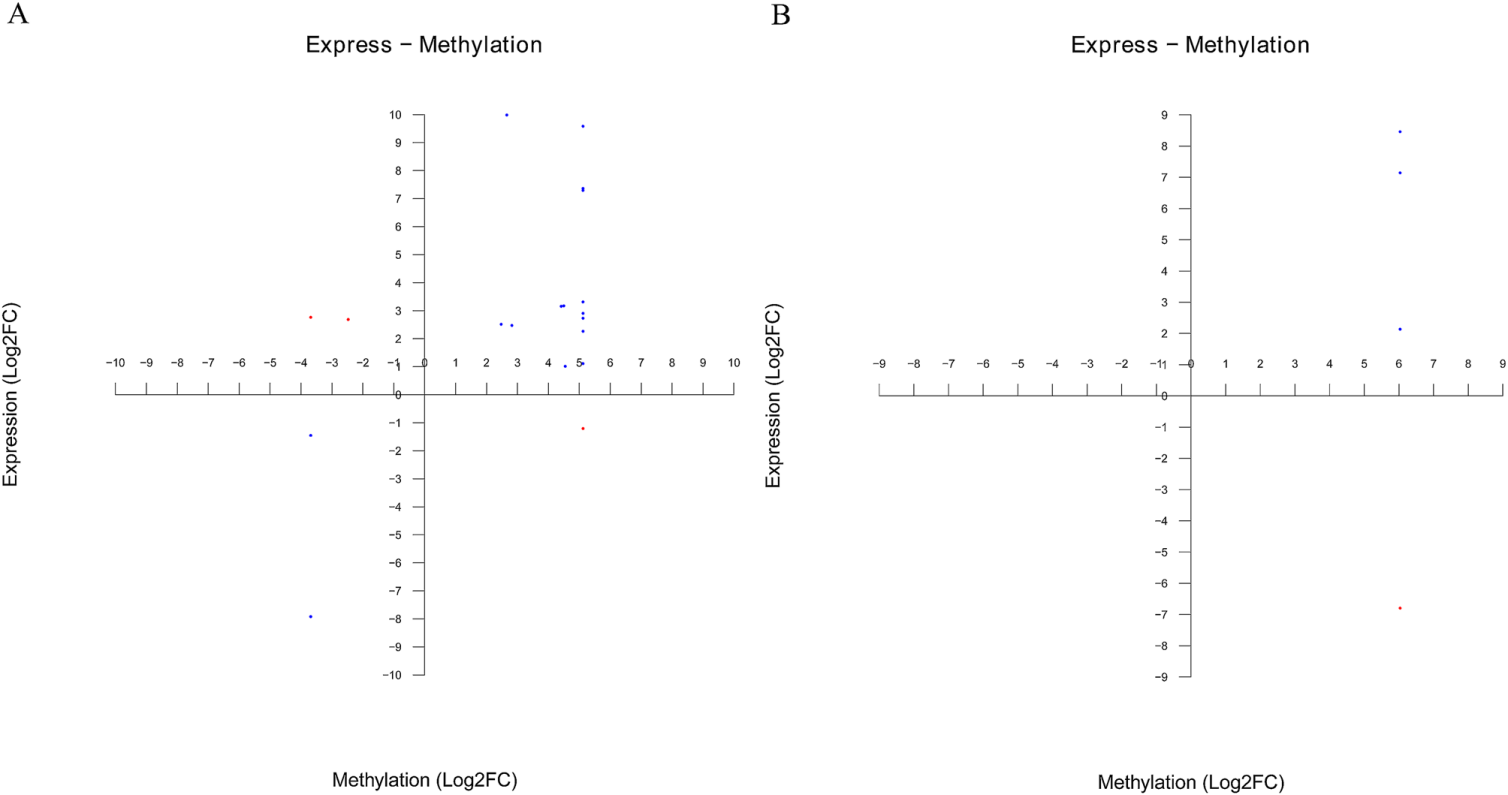
Differentially expressed genes and differential methylation quadrants (**A**: CCGG site; **B**: CCWGG site). Red indicates the locus of negatively correlated differentially expressed genes, and blue indicates the locus of positively correlated differentially expressed genes.

According to the gene names directly related to the genes differentially expressed by DNA methylation and mRNA, a four-quadrant diagram was drawn with the log2 (fold change) of DNA methylation and mRNA. The results showed that most of the genes had high methylation levels and high expression levels, as shown in Fig. 8. A total of 23 related genes were screened by association analysis, in which differential methylation and differential gene expression coincided. Among them, 19 genes were screened at CCGG sites, 16 were positively correlated genes and 3 were negatively correlated genes; 4 genes were screened at CCWGG sites, including 3 positively correlated genes and 1 negatively correlated gene. By comparison with the annotation information, we selected eight positively correlated genes (*Guf1, Col5a1, Kif28p, GPD1, GINS1, CDC7, HPS1,* and *CDK2*) and two negatively correlated genes (*HPS* and *PGM*). These genes will be verified later.

After obtaining the related genes, we carried out GO enrichment analysis on the associated genes. The results showed that 19 associated genes screened at CCGG sites were enriched in 79 terms, 74 of which were significantly enriched (*p*-value < 0.05), including DNA replication, NAD^+^ activity, histidine deaminase activity, and collagen biosynthesis, and the most abundant term was in the cytoplasm. Four associated genes screened at CCWGG sites were enriched in 24 terms, of which 23 were significantly enriched (*p*-value < 0.05), mainly including ATP binding, enzyme activity, and anabolic processes. The annotated associated genes, difference multiples, and GO entries are shown in Table 2.

**Table 2:** Annotated associated genes, difference multiples, and GO entries.

Gene name	Gene ID	log2FoldChange	Pval	GO: ID
*Guf1*	BSL78_23571	9.984866307	1.13E−09	GO:0003924,GO:0005525,GO:0005739,GO:0005743,GO:0005759,GO:0006412,GO:0043022,GO:0045727
*Col5a1*	BSL78_00552	3.154686471	0.000498194	GO:0001568,GO:0003007,GO:0005201,GO:0005581,GO:0005588,GO:0005604,GO:0005615,GO:0007155,GO:0008201,GO:0016477,GO:0030020,GO:0030198,GO:0030199,GO:0031012,GO:0032964,GO:0035313,GO:0035989,GO:0043394,GO:0043588,GO:0045112,GO:0048407,GO:0048592,GO:0051128,GO:0062023,GO:0097435
*Kif28p*	BSL78_16398	2.263659644	0.000680428	GO:0003777,GO:0005524,GO:0005622,GO:0005871,GO:0005874,GO:0007005,GO:0007018,GO:0008017,GO:0016887,GO:0030705,GO:0031966,GO:0072384
*GPD1*	BSL78_02393	1.012943009	0.006624507	GO:0004367,GO:0005975,GO:0009331,GO:0042803,GO:0046168,GO:0051287
*HK1*	BSL78_29015	2.513422691	0.007845006	GO:0001678,GO:0004396,GO:0005524,GO:0005536,GO:0005623,GO:0006096,GO:0019318
*GINS1*	BSL78_01637	3.310931567	0.012114576	GO:0000811,GO:0001833,GO:0005737,GO:0043138,GO:1902983
*CDC7*	BSL78_06196	2.729895734	0.013289412	
*HAL*	BSL78_15654	2.905181661	0.020841636	GO:0004397,GO:0005737,GO:0006548,GO:0019556,GO:0019557
*HEATR4*	BSL78_12133	1.112729419	0.025594608	
*HPS1*	BSL78_19069	− 1.455875532	0.026970908	GO:0005085,GO:0031085,GO:1903232
*Pgm*	BSL78_24377	− 1.206902046	0.041339536	GO:0000287,GO:0004614,GO:0005978,GO:0006006
*CDK2*	BSL78_10932	2.13525817	0.017841031	GO:0004693,GO:0005524,GO:0006468,GO:0007049,GO:0051301

The results of KEGG enrichment analysis showed that *PGM, HK1, GPD1, HAL, CDC7, Col5a1*, and other related genes were enriched in 20 KEGG pathways (*p*-value < 0.05), including glucose metabolism, glycolysis and glycogenesis, lipid metabolism, MAPK signaling pathway, pentose phosphate pathway, carbohydrate digestion and absorption, protein digestion and absorption, cell division and cell cycle, HIF-1 signaling pathway, histidine metabolism, carbon metabolism, purine metabolism, and insulin signaling pathway. The CCWGG associated genes such as *PC* and *CDK2* were enriched in 11 KEGG pathways (*p*-value < 0.05), including the carbon fixation pathway, TCA cycle, pyruvate metabolism, amino acid biosynthesis, cell cycle, carbon metabolism, oocyte meiosis, progesterone synthesis in mature oocytes, p53 signaling pathway, FOXO signaling pathway, and PI3K Akt signaling pathway. These pathways are closely related to individual growth and development.

### Validation of key genes analysis

**Figure 9:**
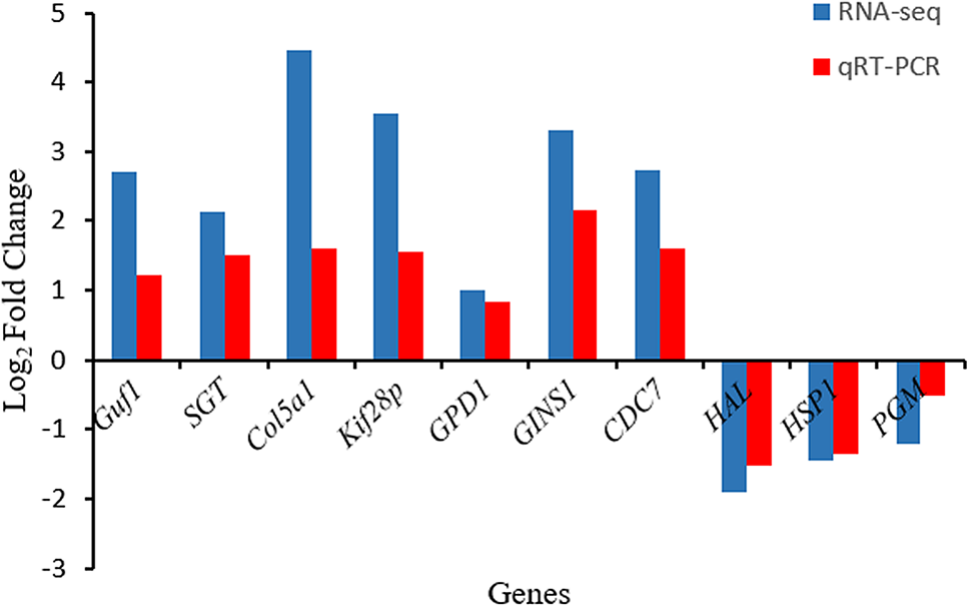
Comparison of mRNA expression levels among the 10 DEGs obtained using qRT-PCR validation and RNA-seq. Log_2_ Fold Change are expressed as the ratio of gene expression after normalization to *Cytb.*

The mRNA levels of *Guf1, SGT, Col5a1, Kif28p, GPD1, GINS1, CDC7, HAL, HPS1,* and *PGM* were detected by qRT-PCR. The results showed that the mRNA levels of *Guf1, SGT, Col5a1, Kif28p, GPD1, GINS1,* and *CDC7* in triploid sea cucumbers were higher than those in diploid sea cucumbers, and the mRNA levels of *HAL, HPS1*, and *PGM* in triploid sea cucumbers were higher than those in diploid sea cucumbers. The results showed that the qRT-PCR results were consistent with the transcriptome sequencing results, which verified that the transcriptional results were credible, as shown in Fig. 9.

In this study, six genes were randomly selected for the pyrophosphorylation sequencing experiment, and the MethylRAD-seq sequencing results were verified. According to a standard regression analysis, the two sequencing results were consistent, and the correlation coefficient is 0.95, as shown in Table 3.

**Table 3:** The result of the pyrophosphorylation sequencing.

	Site1	Site2	Site3	Site4	Site5	Site6
Methylation level	28.58	10.79	10.54	35.14	0.99	12.76
Mutation rate of the pyrophosphorylation sequencing	98.02	89.08	91.64	100	83	91

## Discussion

In recent years, RNA-seq technology has been widely used in biological research, medical research, and drug development. As the technology has developed, it has also been widely used in the aquatic industry. In 2011, Sun et al.^29^ first published the transcriptome data of sea cucumber body wall and intestine tissues, which provided a wealth of data that enabled study of the sea cucumber regeneration mechanism. Du, Zhou and Zhao et al. constructed a transcriptome library of different tissues of sea cucumbers from embryo to adult and in two states (summer and normal), enriched and perfected the sea cucumber transcriptome, and identified a large number of genes related to development, growth, metabolism, and immunity of sea cucumbers^23,24,30^. Research on the polyploidy of aquatic animals has mostly focused on fish and shellfish (oysters). Studies have shown that more than 90% of the liver genes of diploid and triploid bighead fish have a similar expression of unigenes, regardless of the ploidy, and there are 362 up-regulated genes and 83 down-regulated genes, with triploids having at least two-fold more changes relative to diploids^25^. In terms of shellfish, the current researches focus on triploid oysters, triploid *Haliotis discus hannai* and triploid *Patinopecten yessoensis*. The main topics focus on the research and development of induction technology, ploidy detection methods, karyotype and behavior analysis and the applications of polyploidy^31–35^. In this study, transcriptome sequencing was performed on six samples of triploid sea cucumber body wall tissues and diploid sea cucumber body wall tissues, and a total of 314.96 M clean reads were obtained. Changes in gene expression are usually caused by changes in gene copy number, which has been confirmed in yeast, mice, humans, and other species^36^. Data analysis of the FPKM value showed that there was only a small increase in gene expression in triploid sea cucumbers, but there was no significant difference in the number of genes expressed across the six samples, and the correlation between gene expression and gene dose changes was low, thus, we speculated that the triploid involved in diploid has no obvious “dosage effect”, and the gene expression is relatively conservative.

In this study, 640 DEGs were screened from the body wall tissue of triploid sea cucumbers and diploid sea cucumbers by transcriptome sequencing. Through the GO and KEGG enrichment analyses, most of the up-regulated DEGs were found to be related to primary metabolism and secondary metabolism. The enhancement of these basic metabolisms is generally considered to be the basis of the polyploid growth advantage. The up-regulated DEGs were significantly enriched in the pathways of amino acid synthesis, fructose metabolism, and nicotinamide metabolism (*p*-value < 0.05), which indicated that the chromosome increase caused an increase in protein and basal metabolism^37^. At the same time, we also found that the expressions of genes related to cell division, such as *CDC7*, *CDK2*, and *ORC1*, were up-regulated, indicating that chromosome doubling was achieved by controlling cell division^38^. We also found that the expressions of FMS-like tyrosine kinase 1 (*sFLT1*) and C kinase insert domain protein receptor (*KDR*) decreased significantly. These two genes are receptors of vascular endothelial growth factor (VEGF) and antagonists of VEGF^39^. When the expression of these two receptors decreases, cell proliferation is positively affected. Therefore, triploid sea cucumbers grow rapidly, which is the genetic basis of the triploid growth advantage.

MethylRAD-seq technology is a promising research method, and its application in aquatic animals gives very reliable results. In 2017, the latest genome sequencing data of *Apostichopus japonicus* was published^40^, which improved the information on the sea cucumber genome and provided more reference data for the alignment and annotation of methylation sites. In this study, triploid sea cucumbers and diploid sea cucumbers were the research objects. MethylRAD-seq technology was used to study the whole-genome DNA methylation of triploid sea cucumbers. A large number of methylation sites and differentially methylated genes were obtained. According to the results, we found that were significantly more methylation sites of CCGG than of CCWGG in each sample. CCGG methylation sites belong to the CpG type. Therefore, we speculated that methylated cytosine in the triploid sea cucumber genome was mainly located on the CpG dinucleotide, which was consistent with the research results on the scallop (*Patinopecten yessoensis*)^41^. In this study, the distribution of methylation sites in different gene elements in each sample was statistically analyzed. The results showed that the CCGG methylation sites of diploid sea cucumbers were mainly in gene regions, followed by introns, and CCWGG methylation sites were concentrated in the gene interval and exon interval. It has been found that promoter methylation is closely related to transcriptional inhibition. Methylation in the gene region regulates gene expression in contrast to promoter methylation. Methylation in the gene region was shown to be closely related to the transcriptional activation mechanism^42^. In this study, methylation sites in the gene region occurred more frequently in the gene interval, which may mean that methylation in the gene interval can increase the transcription apparatus and enhance the growth and metabolism of triploid *A. japonicus*, and is conducive to biological adaptation to a changeable environment^43^. In recent years, studies have found that DNA methylation is related to intron repeat element silencing, and exon DNA methylation may be related to RNA selective splicing^44^. Therefore, there are DNA methylation sites in the exon and intron regions of diploid and triploid sea cucumbers.

In this study, 615 differentially methylated genes were screened at CCGG sites, including 325 up-regulated genes and 190 down-regulated genes; 447 differentially methylated genes were screened at CCWGGA sites, including 292 up-regulated genes and 155 down-regulated genes. Through the GO enrichment analysis of differentially methylated genes, we found that they were significantly enriched in cell number, DNA, RNA synthesis, and enzyme activity. This may be due to the rapid growth of triploid sea cucumbers, as cell division produces a large amount of newly replicated DNA, and DNA methylation is activated and expressed by genome doubling to maintain genomic stability. Through the KEGG enrichment analysis of methylated differentially expressed genes, the results showed that differentially methylated genes at CCGG sites were significantly enriched in the pentose phosphate pathway, cell cycle, and disease; differentially methylated genes at CCWGG sites were significantly enriched in virus infection and glycerophosphate metabolism, and the PI3K Akt signaling pathway was the most abundant pathway. Studies have shown that the size of individual differences has a great impact on glucose metabolism. The sea cucumber is large and grows fast, and its glucose metabolism and energy metabolism are enhanced. Most of the known genes involved in the glucose metabolism pathway are directly affected by the epigenetic mechanism. In mice, the sensitivity of liver tissue to diet-induced obesity and insulin resistance is related to the methylation of insulin-like growth factor binding protein 2 (IGFBP2)^45^. In juvenile rainbow trout, a carbohydrate-rich diet has an effect on the DNA methylation level of the glucose-6-phosphatase CpG site in liver tissue involved in gluconeogenesis^46^. Some studies have shown that the body wall of the sea cucumber has a strong regenerative ability^29,47^. During wound healing, as the wound heals, the content of fibronectin in the wound increases^48^. The binding of IGF-1 with IGF-1 receptor can activate the PI3K / Akt signal pathway, interfere with cell apoptosis^49^, induce the expression of cell growth factor, enhance collagen secretion, and promote faster healing of sea cucumbers. The analysis of the metabolic pathway and differentially methylated genes showed that there were significant differences in the growth and nutrient metabolism pathways of diploid sea cucumbers compared to triploid sea cucumbers.

DNA methylation is closely related to the regulation of gene expression, but the relationship between DNA methylation and the gene expression level is more complex. The methylation of promoter DNA is closely related to the degree of gene transcription inhibition. Through the analysis of the transcriptome data of sea cucumber tissue, it was found that there was a weak positive correlation between the gene methylation level and the transcription level^50^, which was consistent with previous research results on gene methylation of the oyster^51^. In addition, DNA methylation can inhibit the transcription of some transposons and promote gene expression^52^. Our analysis also showed that the methylation of triploid and diploid sea cucumber genes mainly occurred in the gene region. Through these results, we speculated that DNA methylation of aquatic animals may play a role in promoting gene expression. In this study, we analyzed the association between differentially methylated genes and differentially expressed genes. We found that 19 differentially methylated genes were also differentially expressed genes, indicating that these 19 genes may be regulated by DNA methylation. Through the KEGG enrichment analysis of these 19 genes, we found that they were mainly enriched in the basic metabolic pathways such as glucose metabolism, lipid metabolism, histidine metabolism, carbon metabolism, purine metabolism, protein digestion and absorption, as well as cell division and cell cycle, MAPK signaling pathway, HIF-1 signaling pathway, insulin signaling pathway, p53 signaling pathway, FoxO signaling pathway, and PI3K Akt signaling pathway, which are related to growth and development. These pathways play an important role in the growth and development of individuals, indicating that DNA methylation plays an important role in regulating the growth and development and energy metabolism of the triploid sea cucumber. Cyclins/Cdks protein kinases play a key role in cell cycle regulation, in which Cyclin E/CDK2 participates in the Gl/S test point conversion of the cell cycle^53^. DBF4B and CDC7 kinase jointly participate in and regulate the initiation and progress of DNA replication, and play an important regulatory role in the cell cycle^54^. Studies have found that *HPS1* and *PGM* genes are negatively correlated genes, and *HPS1* is related to the abnormal structure and function of some organelles (melanosomes, platelet dense granules, and lysosomes)^55^. In energy metabolism, PGM catalyzes glucose-1-phosphate to produce glucose-6-phosphate in humans. This reaction is reversible. We speculate that these genes affect the cell proliferation and differentiation of triploid sea cucumbers under the regulation of DNA methylation and energy metabolism, thereby affecting the growth and development of triploid sea cucumbers. Considering that the regulatory relationship between DNA methylation and gene expression is still controversial^56^, we will conduct more in-depth research on the relationship between DNA methylation and gene expression in sea cucumbers in the future.

## Conclusion

In this study, RNA-seq and MethylRAD-seq were used to analyze the transcriptome and DNA methylation of the body wall tissue of triploid and diploid sea cucumbers. We constructed the gene expression profile of the body wall tissues of triploid sea cucumbers and preliminarily speculated that the growth advantage of the triploid sea cucumber was not related to a “dosage effect”. We constructed a DNA methylation map of triploid sea cucumbers and, combined with transcriptome analysis, differentially expressed genes that were also differentially methylated were screened. Since the screened differentially expressed genes were related to growth and metabolism, we speculated that genomic DNA sequence variations and changes in methylation levels and genetic pattern changes occurred to a certain extent during the polyploidy of sea cucumbers. We believe that the dominance of triploid sea cucumbers is related to genome variation and the readjustment of DNA methylation patterns. In the future, we will continue to investigate the molecular mechanism of the growth advantage of triploid sea cucumbers, systematically cultivate and raise triploid sea cucumbers, and analyze the transcriptome and methylation of other tissues of triploid sea cucumbers, such as the gonads, respiratory trees, and longitudinal muscles.

## Materials and methods

### Diploid and triploid sea cucumber samples

Sea cucumbers (*A. japonicus*) used in this study came from the Key Laboratory of Marine Aquaculture in Northern China, Ministry of Agriculture, and rural areas. Polyploid sea cucumbers (*A. japonicus*) were obtained by using the optimized hydrostatic pressure method in 2017^4^. Sperm and eggs were collected within 0.5 h after spawning of the female sea cucumber, and single female sea cucumber eggs were fertilized in 21 °C water. Hydrostatic pressure induction was conducted 7 min after fertilization, with the pressure set at 65 mPa and the treatment lasted for 5 min. Fertilized eggs were then transferred to the hatching water body. After optimization, the fertilization rate reached 98%, and the highest hatching rate was as high as 76%.

To verify the successful induction of triploid sea cucumbers, 1.2 ml of cell lysate (cystatin UV precise P/05-5002, Japan) and a small number of tube feet were added to a 1.5-ml centrifuge tube. After being disrupted, the cellular components were analyzed by flow cytometry (Sysmex, Japan). Then, the triploid sea cucumbers and diploid sea cucumbers were cultured under the following conditions: 16 ± 1.5 °C, salinity 30 ± 1, and pH 7.0. During the breeding process, the water was changed every two days and food was provided (feed formula: sea mud, compound feed, and spirulina powder). The study was carried out in April 2019. The body wall tissues of three diploid sea cucumbers (100 ± 15 g) and three triploid sea cucumbers (105.66 ± 50 g) were collected and frozen in liquid nitrogen and stored in a refrigerator at −80 °C.

### RNA-seq library construction, sequencing, and data analysis

Total RNA was extracted using the mirVana miRNA Isolation Kit (Ambion, Texas, USA) following the manufacturer’s protocol. RNA integrity was evaluated using the Agilent 2100 Bioanalyzer (Agilent Technologies, Santa Clara, CA, USA)^57^. Samples with RNA integrity number (RIN) ≥ 7 were subjected to subsequent analysis. The libraries were constructed using TruSeq Stranded mRNA LTSample Prep Kit (Illumina, San Diego, CA, USA) according to the manufacturer’s instructions. Then these libraries were sequenced on the Illumina sequencing platform (HiSeqTM 2500 or Illumina HiSeq X Ten), and 125 bp/150 bp paired-end reads were generated^58^.

The transcriptome sequencing and analysis were conducted by OE Biotech Co., Ltd. (Shanghai, China). Raw data (raw reads) were processed using Trimmomatic^59^. Reads containing ploy-N and low-quality reads were removed to obtain the clean reads. Then the clean reads were mapped to the reference genome (GCA_002754855.1) using hisat2^60^. The fragments per kilobase of exon per million reads mapped (FPKM)^61^ value of each gene was calculated using cufflinks^62^, and the read counts of each gene were obtained using HTSeq-count^63^. DEGs were identified using the DESeq R package functions^64^ “estimateSizeFactors” and “nbinomTest”. A *p*-value < 0.05 and Fold Change > 2 or Fold Change < 0.5 were set as the thresholds for significantly differential expression. A hierarchical cluster analysis of DEGs was performed to explore the gene expression patterns. GO enrichment and KEGG^65^ pathway enrichment analysis of the DEGs were performed using Perl scripts in R based on the hypergeometric distribution. The alternatively splicing analysis of differentially regulated transcripts, isoforms, or exons was performed using ASprofile^66^. SNP and INDEL were called using SAMtools^67^ and BCFtools^68^; the details are shown on the SAMtools webpage (http://samtools.sourceforge.net/mpileup.shtml). Then SnpEff^69^ annotated and predicted the effects of the variants on the genes.

### DNA methylation library construction, sequencing, and data analysis

The CTAB method was used to extract six samples of DNA. The integrity of the DNA was detected by 1% agarose gel electrophoresis. There was a complete strip. If there was no towing, the purity and concentration of the samples were detected by the NanoPhotometer nucleic acid protein analyzer (Germany). After they were quantified, the DNA samples were stored at − 80 °C. Libraries were constructed using MethylRAD-seq technology and high-throughput sequencing was performed on the Illumina SE sequencing (50 bp) platform.

Then, the original data obtained from sequencing were subjected to quality control. If more than 15% of base pairs had low-quality values or too many N bases in the obtained reads, they were deleted. Through SOAP software (parameter settings: –M = 4, –v = 2, –r = 0), enzyme reads were compared to the reference genome (GCA_002754855.1) to find reliable methylation sites. According to the annotated information, SnpEff software (version: 4.1G) was used to obtain the UTR region, and BEDTools software was used to calculate the distribution of methylation sites in the different gene elements in the six samples; the *p*-value and Log_2_FC of each locus were calculated using edgeR software. According to the sequencing depth of each locus in the six samples, the methylation levels of the two groups were compared; genes with *p*-value ≤ 0.05 and |Log_2_FC|> 1 were screened, and their methylation levels and annotation information were sorted out. Finally, GO and KEGG enrichment analyses were performed on the differential genes.

### Association between the transcriptome and DNA methylation in the body wall of triploid sea cucumbers

According to the data on mRNA and MethylRAD expression and relative content, the Pearson test was used to calculate the correlation between gene expression and DNA methylation. Go and KEGG enrichment analyses were carried out on the selected related genes to describe their functions and the affected pathways.

### Pyrophosphate methylation sequencing and qRT-PCR verification of the differential genes

Six differential genes were randomly selected from the MethylRAD sequencing results, and common PCR primers and the pyrophosphate amplification primers were designed. Samples for Methyl RAD sequencing were placed in a bisulfite amplification kit, and the methylated sequencing results were verified by PCR amplification of biotin-labeled products. The Trizol method was used to extract total RNA from the body wall tissues of triploid and diploid sea cucumbers. The RNA was reverse transcribed into cDNA, and *Cytb* was used as an internal reference gene for real-time fluorescence quantitative PCR amplification. The PCR primers were designed and synthesized by Shanghai Sangon Biotech and the primer sequences are shown in Table S4.

## Supplementary Information

Supplementary Information

## Data Availability

The datasets generated and analysed during the current study are available from the corresponding author on reasonable request.
