# Supplementary material for: Analysis of the gene transcription patterns and DNA methylation characteristics of triploid sea cucumbers (Apostichopus japonicus)
Source: Sci Rep. 2021 Apr 7;11:7564. doi: 10.1038/s41598-021-87278-9 (PMC8027599; doi:10.1038/s41598-021-87278-9)
Supplement: Supplementary file 1 — Supplementary Information [file 41598_2021_87278_MOESM1_ESM.doc]

**Supplementary Information**

Table S1. Sequencing data quality processing results

| Sample | raw_reads | raw_bases | clean_reads | clean_bases | valid_bases | Q30 | GC |
| --- | --- | --- | --- | --- | --- | --- | --- |
| 2N_1 | 49.13M | 7.37G | 47.75M | 6.94G | 94.16% | 95.49% | 39.96% |
| 2N_2 | 56.72M | 8.51G | 52.97M | 7.62G | 89.60% | 91.94% | 40.20% |
| 2N_3 | 53.42M | 8.01G | 51.06M | 7.42G | 92.59% | 93.15% | 40.45% |
| 3N_1 | 57.13M | 8.57G | 55.18M | 8.01G | 93.52% | 93.47% | 39.30% |
| 3N_2 | 58.12M | 8.72G | 56.08M | 8.17G | 93.69% | 93.37% | 42.60% |
| 3N_3 | 53.77M | 8.06G | 51.92M | 7.54G | 93.48% | 93.40% | 42.36% |

Table S2. Alignment rates between reads and the reference genome

| Sample | 2N-1 | 2N-2 | 2N-3 | 3N-1 | 3N-2 | 3N-3 |
| --- | --- | --- | --- | --- | --- | --- |
| Total reads | 47748998 | 52971580 | 51058546 | 55179170 | 56078006 | 51917842 |
| Total mapped reads | 20454928(42.84%) | 34010781(64.21%) | 33201354(65.03%) | 36203270(65.61%) | 41985398(74.87%) | 39596210(76.27%) |
| Multiple mapped | 2054573(4.30%) | 3170080(5.98%) | 3199227(6.27%) | 4009773(7.27%) | 3713994(6.62%) | 3184238(6.13%) |
| Uniquely mapped | 18400355(38.54%) | 30840701(58.22%) | 30002127(58.76%) | 32193497(58.34%) | 38271404(68.25%) | 36411972(70.13%) |
| Read-1 | 9217362(19.30%) | 15403514(29.08%) | 14998194(29.37%) | 16112942(29.20%) | 19166745(34.18%) | 18216189(35.09%) |
| Read-2 | 9182993(19.23%) | 15437187(29.14%) | 15003933(29.39%) | 16080555(29.14%) | 19104659(34.07%) | 18195783(35.05%) |
| Reads map to '+' | 9251473(19.38%) | 15528573(29.31%) | 15012772(29.40%) | 16171548(29.31%) | 19210569(34.26%) | 18236207(35.13%) |
| Reads map to '-' | 9148882(19.16%) | 15312128(28.91%) | 14989355(29.36%) | 16021949(29.04%) | 19060835(33.99%) | 18175765(35.01%) |
| Non-splice reads | 13156462(27.55%) | 20895847(39.45%) | 20010679(39.19%) | 23034537(41.74%) | 21984529(39.20%) | 21105780(40.65%) |
| Splice reads | 5243893(10.98%) | 9944854(18.77%) | 9991448(19.57%) | 9158960(16.60%) | 16286875(29.04%) | 15306192(29.48%) |
| Reads mapped in proper pairs | 16731804(35.04%) | 27420650(51.76%) | 26533032(51.97%) | 28939476(52.45%) | 35112220(62.61%) | 33729382(64.97%) |

Table S3. Sample sequencing data volumes and comparison rates

| Sample | Raw Reads | Enzyme Reads | Mapping Reads | Ratio |
| --- | --- | --- | --- | --- |
| 2N-1-1 | 30557096 | 16001928 | 7350917 | 45.94% |
| 2N-2-1 | 30264548 | 18224276 | 8224942 | 45.13% |
| 2N-3-1 | 30002105 | 15474909 | 7210883 | 46.60% |
| 3N-1-1 | 30162255 | 15319070 | 7161516 | 46.75% |
| 3N-2-1 | 30262519 | 15834732 | 7363641 | 46.50% |
| 3N-3-1 | 30710783 | 17305997 | 7972814 | 46.07% |

Table S4. Primer sequences

| Primer names | Sequence (5'→3') | Purpose |
| --- | --- | --- |
| 1 | 1-F：AGGGTTTATTATAGGGGAAAAGG  1-R：CACAACTTCCAATAAACCATCCATAAAA  1-S：AAGATATTGTTATTGGTTAGAAA | Pyrophosphate sequencing |
| 2 | 2-F：TTGGTATGTTATATTAGGAGTTTGTTAT  2-R：CATTTCATCCAATCAACTCAATCTA  2-S：GTTAATGTGTTTGTTGTTAGT | Pyrophosphate sequencing |
| 3 | 3-F：TGAATGGATTTGGTTAAGTTTTGATGAA  3-R：ACTCAATCATTTATATCACCCTATCT  3-S：TGTAAATTTGATGTAAATGTAAAG | Pyrophosphate sequencing |
| 4 | 4-F：TTTTGTGGAGGAGAGTGTATAGATGT  4-R：ACACTTTAAATCTTATCATACCACACTTC  4-S：ATAATAAATATATCAATCTTACCTC | Pyrophosphate sequencing |
| 5 | 1. F：GGGTTGGGTTGTTTGTTAATG   5-R：CTAAAACCCCCAAACTTAAACAATACCAA  5-S：GGTGAAAAGGTGTAGG | Pyrophosphate sequencing |
| 6 | 6-F：TAGTTGTGGATATGTGGGTAGT  6-R：ACAACCATCTTAACCTCCTCTA  6-S：AGGTATTTATTTTTTAAGGTTTTAG | Pyrophosphate sequencing |
| *AjGuf1*-F | AAGTCCAGGGAAGTTGGCAAAGC | qRT-PCR |
| *AjGuf1*-R | TGCTACCGATGGCTGCTTGTATTG | qRT-PCR |
| *AjCol5a1*-F | CGGAGGAGGAGGAGGAGTCTTTC | qRT-PCR |
| *AjCol5a1*-R | ACCATGTGTCCGTCTGTCTGTTTG | qRT-PCR |
| *AjKif28P*-F | ACGGGTGCTACAGGTGACAGG | qRT-PCR |
| *AjKif28P*-R | GCCAGTGCTGCGATACAGTTACC | qRT-PCR |
| *AjGPD1*-F | CTGTAATCCGCCTTGGACTCATGG | qRT-PCR |
| *AjGPD1*-R | ACGCCGCAACTCTCTAAGAATGTG | qRT-PCR |
| *AjGINS1*-F | CAATGAGGTGGGAGTTTGGGAGTG | qRT-PCR |
| *AjGINS1*-R | AGGAGGCCAGTGATTTGCTGTAAC | qRT-PCR |
| *AjCDC7*-F | AGAGGAGGTCAAGATGGCTGCTAG | qRT-PCR |
| *AjCDC7*-R | GTGTGGAGACTGGTGGTAGAGGAG | qRT-PCR |
| *AjHPS1*-F | GCGTGCCTTAGTAGTCGTCTGC | qRT-PCR |
| *AjHPS1*-R | CCTTCCTGTTGCCATCCACTCTG | qRT-PCR |
| *AjHAL*-F | ACAGCAGCCTCCCTTGTCTCAG | qRT-PCR |
| *AjHAL*-R | AATGACTTCCTGGCAGCGAATCC | qRT-PCR |
| *AjPGM*-F | GCAGCAGCCAATCAGGTGAGG | qRT-PCR |
| *AjPGM*-R | GTGAGTATAATGCCGCCGGTAGC | qRT-PCR |
| *AjCDK2*-F | CGAGTCACGACAAGTCAGAACGAG | qRT-PCR |
| *AjCDK2*-R | CTGTTGCTGCTGTTGCTGATGATG | qRT-PCR |
| *AjSGT*-F | CGCCGCAGCTCGAATCTTCC | qRT-PCR |
| *AjSGT*-R | TGACCCTGATCGCCATTGAAATCG | qRT-PCR |
| *AjCRADD*-F | TTCACAGGAGAGCCCTAGACAAGG | qRT-PCR |
| *AjCRADD*-R | AGCGGTTCTGGTTGGTATTGCATC | qRT-PCR |
| *Cytb*-F | TGAGCCGCAACAGTAATC | qRT-PCR |
| *Cytb*-R | AAGGGAAAAGGAAGTGAAAG | qRT-PCR |
